# Total product lifecycle regulatory considerations and recommendations for generative AI-enabled medical devices

**DOI:** 10.1093/ehjdh/ztag019

**Published:** 2026-02-03

**Authors:** Antonis A Armoundas, Jagmeet P Singh

**Affiliations:** Cardiovascular Research Center, Massachusetts General Hospital, 149 13th Street, Charlestown, MA 02129, USA; Broad Institute, Massachusetts Institute of Technology, Cambridge, MA 02139, USA; Cardiology Division, Cardiac Arrhythmia Service, Massachusetts General Hospital, Boston, MA 02215, USA

**Keywords:** Regulation, Policy, Generative artificial intelligence, Large language models, Performance, Equity, Health care

## Abstract

As generative artificial intelligence (GenAI) emerges within the healthcare ecosystem, the regulatory environment surrounding these technologies remains fragmented. Importantly, GenAI in healthcare requires adapting the established Total Product Lifecycle (TPLC) paradigm to non-deterministic, rapidly evolving software, translating it into concrete steps for pre-market evaluation, change controls, and post-market performance monitoring to keep devices safe and effective as they change. This manuscript elucidates the need for developers, healthcare providers, and clinicians to engage proactively with regulatory compliance during the development and deployment phases of genAI-enabled medical devices. By understanding the nuances of national and international regulations, stakeholders can better navigate the unique risks of the evolving landscape of GenAI-enabled medical devices, while ensuring patient safety and outcome optimisation. Finally, this manuscript highlights the importance of the TPLC approach in managing the risks associated with GenAI-enabled technologies, offering recommendations for improving health outcomes and balancing the interests of various stakeholders.

## Graphical Abstract

**Graphical Abstract:**
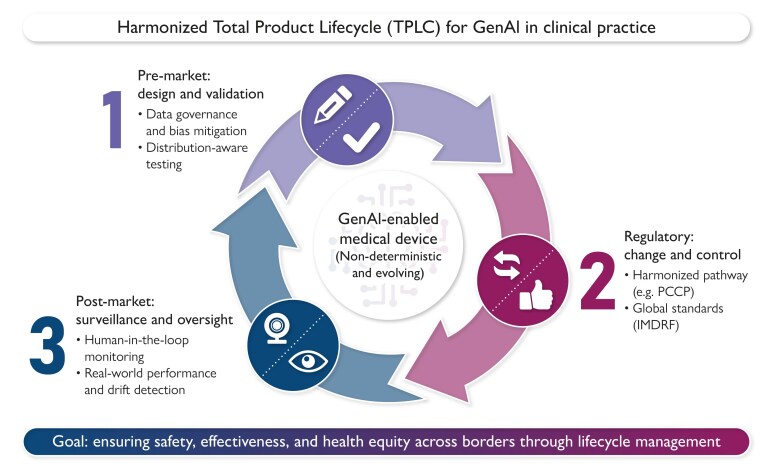

## Introduction

As digital health technologies^1^ and artificial intelligence (AI)^2^ are transforming medical research, health care, and public health, the ever-increasing usage of algorithms in health care has challenged national governments,^3–8^ regulatory agencies,^9,10^ health organisations,^1,11–13^ developers,^14,15^ and providers. In the advent of generative AI (GenAI), the even closer relationship between GenAI-driven decision-making systems and humans creates new and more complex circumstances, with multiple and possibly competing interests.^16^

This Perspective proposes a GenAI-adapted Total Product Lifecycle (TPLC) framework for developers, healthcare providers, and clinicians. We aim to convert TPLC principles into an operational checklist for GenAI-enabled devices, covering data governance and distribution-aware performance claims in pre-market evaluation; predetermined change control plans (PCCP) based updates with predefined acceptance criteria; and auditable logging, human-in-the-loop controls, and drift surveillance in post-market monitoring, while mapping risks and residual gaps where standards or guidance are still evolving.

### Distinction between non-generative and generative AI

Non-generative (discriminative) AI models learn the conditional mapping from inputs to targets, and optimize a supervised loss function (e.g. cross-entropy for classification, squared error for regression). They select among predefined outcomes, prioritize calibration and decision boundaries, and are evaluated by predictive metrics (i.e. area under the receiver operating characteristic curve).

GenAI models learn the data distribution itself by maximising a likelihood (or an equivalent surrogate) and then sample from that distribution to synthesize new instances.

### GenAI and the total product life cycle

GenAI is defined as the class of AI models that mimic the structure and characteristics of input data in order to generate new, derived synthetic content.^17^ GenAI has the potential to transform workflows, enhance patient experiences, and improve clinical outcomes.

GenAI-enabled medical device refers to a device [a term is defined in section 201(h) of the Federal Food, Drug, and Cosmetic Act], in which GenAI methods or models are integral to the device’s output or functionality. In other words, for GenAI-enabled devices, GenAI methods or models play a critical role in the device’s primary function(s) or directly enable its output(s). Additionally, GenAI-enabled devices are not monolithic and can have a variety of intended uses,^17^ where conventional provisions and regulations may not be generally applicable to all GenAI-enabled devices. Consequently, traditional AI regulatory frameworks may not adequately address the nuances of GenAI-enabled medical technologies.

The International Medical Device Regulators’ Forum (IMDRF) defines ‘Software as a Medical Device (SaMD)’, as software intended to be used for one or more medical purposes without being part of medical device hardware.^18^ Subsequently, the Food and Drug Administration (FDA) has put forward policies that are tailored for SaMD to ensure that technologies that reach users, including patients and healthcare professionals, are safe and effective.^19,20^

The FDA has for a long time promoted a total product life cycle (TPLC) approach to the oversight of medical devices, including AI-enabled devices. Also, the FDA has committed to advancing regulatory approaches for these devices using current regulations, involving the traditional pre-market evaluation and post-market monitoring processes, as well as exploring options that may require new regulations.^9,10^ Such an approach has an increasingly more critical role for modern medical devices that incorporate technologies intended to iterate faster and more frequently over a device’s life of use than ever before.

Contrary to non-generative AI,^2,11^ GenAI-enabled medical devices can be intended to or inadvertently provide variable outputs for the same inputs. Furthermore, these devices may frequently rely on models that are meant to evolve rapidly and often, autonomously or not, being trained on unknown datasets employing non-traditionally established performance AI metrics,^2,11^ and may query models that are not themselves medical devices, offering unique capabilities, creating new and unique risks compared to non-generative AI, requiring new risk-mitigating approaches and specific regulatory challenges. Because GenAI devices produce non-deterministic, state- and context-sensitive outputs, regulators must shift from one-time, point-estimate evaluations to continuous PCCP-based change management, enhanced logging and auditing, throughout the TPLC. Therefore, while these features of GenAI mandate a new regulatory framework of GenAI-enabled medical devices, the FDA’s TPLC approach is likely to remain relevant and play a significant role in the management of future, safe and effective GenAI-enabled medical devices.

## Pre-market evaluation

Recognizing that not every GenAI-enabled technology is a medical device (e.g. software that facilitates hand off from one shift to the next, compared to GenAI-enabled medical devices that provide patients and clinicians specific recommendations for queries and disease management), foundation models leveraged by GenAI-enabled devices are expected to evolve over time, and there may be limited information available on the training data utilized for these pre-trained models, the FDA has identified key challenges aiming to understand the critical information and practices needed for a comprehensive approach to manage risk throughout the TPLC for GenAI-enabled devices, by evaluating the safety and effectiveness of the GenAI-enabled devices during pre-market evaluation.^21^

Performance metrics will typically vary with device intended use and be modality-specific (**Box 1**). Also, performance evolves as the model and the data evolve, including at the place of deployment and its users. In this case, pre-market evaluation involves either stand-alone testing or comparative effectiveness.

Box 1 Considerations and Recommendations for Pre-Market Evaluation of GenAI-Enabled Medical Devices

**Table:** 

***Device Characterization***	define the setting in which the intended GenAI-enabled device will be deployed, its intended use (‘label’), and the targeted populations
	clarify whether models operate autonomously or require human intervention, which would dictate different requirements with respect to transparency and reliability of the GenAI device outputs
***Metrics***	develop domain-specific, intended-use metrics
	develop methods for bias assessment and mitigation
	determine the accuracy of GenAI-produced output, compared to the clinical guidelines
***Data Requirements***	ensure that the genAI-enabled device has been trained and validated in a population that is representative of the target population for use
	monitor and improve patient adherence during a clinical trial
	create standardized pro-forma datasheets that detail data characteristics, including demographics, size and type of the data, and intended use scenarios
	include diverse data sets for training, validating, and improving GenAI models to ensure equitable healthcare outcomes
	access benchmarking data for assessment of equivalence to a prior device
***Adaptation***	promote the continuous training of the GenAI-enabled devices, in a manner that is aligned with the shifting landscape of clinical practice and natural language processing technologies
	create watermarked generated content
***User Training***	create training programs to educate users about the limitations and functionalities of generative GenAI tools, in balancing the demand for efficiency with patient safety
***Usage***	assess and mitigate GenAI outputs resulting in variability in clinician decision-making and patient care
	differentiate between a product change control plan and a usage change control plan
***Accountability***	determine the level of responsibility of the developers, the manufacturing company (e.g. with respect to the reported claims of the GenAI-enabled technology) and the deployers of the technology in a clinical environment
***Pre-Deployment***	perform preclinical deployment validation at a site, with site data, to assess performance at the site
	perform fine-tuning locally, at the site level, in order to get the highest accuracy

However, since GenAI-enabled devices do not act on a human, but on data that came from a device that was attached to a human, the data are continuously changing because the devices that obtain these data are different and are changing, performance cannot be assessed on prior data, as drifting will make impossible the ‘equivalence’ of a GenAI device with another device, at a prior time point (**Box 1**).

## Post-market monitoring

Post-market strategy monitoring starts with pre-market design. The complex landscape in which GenAI models are being deployed in healthcare environments underscores the emphasis needed to be placed on determining and establishing monitoring capabilities, methodologies and metrics to effectively monitor and evaluate the post-market performance of GenAI-enabled devices, through risk management strategies, user training, accountability, and continuous monitoring to ensure safety and effectiveness.

As with traditional AI-enabled devices, responsibility lies across all stakeholders,^14^ with primary responsibility in using a GenAI-enabled device being ‘as labelled’ (**Box 2**). For example, in a proactive action, OpenAI has placed a disclaimer for ChatGPT that it is not a medical device. However, in both the US^9^ and the EU,^22,23^ intended use/purpose, as evidenced by labelling, claims, and functionality, determines whether software is a medical device; vendor terms or disclaimers do not determine if the product is promoted or functions for diagnosis, mitigation, treatment, prevention, or similar medical purposes.^9^ In practice, if the function produces or prioritizes patient-specific diagnostic/therapeutic recommendations, it is typically a medical device; if it organizes or summarizes information for a human who can independently verify its validity, it is typically not.^9^

Box 2 Considerations and Recommendations for Post-Market Monitoring of GenAI-Enabled Medical Devices

**Table:** 

***Intended Use***	use ‘as labelled’, remains a cornerstone for the proper use of GenAI-enabled devices
***Stakeholder Engagement***	engage a diverse range of stakeholders, including patients, in order to understand the impact of GenAI-enabled devices on healthcare delivery and quality
***Type of Monitoring***	perform quantitative vs. qualitative (what does the user want to see) monitoring
	align monitoring with the shifting landscape of clinical practice; incorporation of real-time user feedback is a critical component of this development process
	use of watermarked generated outputs for continuous monitoring of GenAI-enabled devices
	perform stochastic sampling, re-identification, and post-market evaluation by the manufacturer, again
***Establish new Definitions***	establish clear, new definitions around errors and adverse events that are associated with GenAI-enabled outputs, which will be employed for monitoring and regulatory compliance
***Data Accessibility***	access to diverse data sets is essential for training, validating, and improving GenAI models to ensure equitable healthcare outcomes
	access to benchmarking data for performance assessment with respect to a prior time-point
***GenAI Specific Metrics***	establish specific metrics that monitor accuracy, equity, fairness, bias and relevance of GenAI outputs; monitoring gaps in representation and performance across demographic groups is crucial, along with developing robust methodologies for real-world applicability

In addition to the non-deterministic nature of GenAI, key challenges in evaluating the safety and effectiveness of GenAI-enabled devices include their undergoing of continuous change resulting from live localized data, user interactions, and changing conditions (i.e. change in technologies acquiring the data, patient demographics, etc).

In addition, there is an increased need for transparency and explainability with respect to the training data, as well as performance variability evaluation during post-market monitoring; for example, by employing confidence intervals to demonstrate the reliability of outputs (outcomes), whereas high variability should increase the post-market monitoring (**Box 3**). A human expert in the loop is necessary to evaluate the safety, effectiveness and performance variability of the GenAI-enabled device.

Box 3 Recommendations for Post-Market Monitoring of GenAI-Enabled Medical Devices (continued)

**Table:** 

***Monitoring Capabilities***	promote decentralized studies to allow for the local evaluation of the GenAI-enabled devices
	establish internal periodic reviews of GenAI outputs and clinician interactions
	establish reporting systems that include watermarks to identify AI-generated outputs
	establish processes that ensure equitable access in order that diverse populations are represented in GenAI training data, and avoid biases and disparities in healthcare outcomes
	promote training, user engagement and patient education; place emphasis on the necessity of training patients and clinicians on understanding GenAI outputs; engage stakeholders in co-creating educational processes that will increase usability and trust in GenAI technologies
	create training and user engagement feedback loops; establish efficiencies and feedback mechanisms to gather user experiences and improve model performance, continuously
	employ federated post-market monitoring and evaluation
	assess performance of subgroups, including by postal code; examine the application of the regulatory purview of claims by manufacturers
***Error Reporting Mechanism***	establish error reporting processes for manufacturers and users (clinicians and patients), with respect to accuracy, safety and boundness of outcomes
	establish a centralized reporting mechanism in a database or registry, for transparently reporting GenAI errors and monitoring performance to help identify patterns, and drive improvements in GenAI applications
	establish benchmarks for GenAI-enabled devices against traditional AI and pre-existing medical devices to assess their operational effectiveness and safety
	establish benchmark data for continuous performance evaluation of GenAI-enabled devices
	employ ‘I have not seen this before’ type statements of GenAI-enabled devices, to help both clinicians and developers in further training the models in those areas
	establish of local error adjudication boards
	determine whether failure was due to product failure or Gen-AI algorithm implementation failure
***Outcome Assessment***	establish clinical outcomes
	assess patient satisfaction
	assess quality of care
	assess health equity

It is evident that an actionable GenAI-specific framework is needed to ensure GenAI technologies are safe and enhance healthcare quality. In this effort, which focuses on the continuous iteration and improvement of GenAI systems, the collaboration among regulatory bodies, healthcare providers, and technology developers is of critical importance and necessary.

## Risks and risk management

The promise of GenAI-enabled medical devices should also recognize the need for rigorous assessment protocols to adequately understand risk (**Box 4**).^10^

Box 4 Understanding Risk

- categorize the output of GenAI devices according to risk
- recognize that risk changes according to the type of input data, type of model, and type of output (outcome) data
- the training data are not known, so foundational models cannot be assessed for error rate or drift
- when new algorithms are trained on prior GenAI-produced reports, error accumulates
- when algorithms are learning from retrospective data that no longer reflect operative health care practice policies, and are evaluated in present time health care practice policies (‘off-policy AI learning’), they result in performance drift
- failure modes determine clinical harm and risk, establish, and assess residual risk of mitigation measures
- establish patient- vs. clinician-user risk, and associated liability and accountability
- despite imposing guardrails, one should be mindful of ‘human drift’, on how, for example, these GenAI devices are used, especially when off-label use is attempted
- establish the ability of people to understand ‘GenAI-device labels’
- use of benchmarking data may allow for the continuous assessment and mitigation of drift
- benchmarking also involves assessment of capabilities and performance, beyond risk; users should be educated about potential limitations and errors, and trained (‘human in the loop’) to assess performance both quantitatively and qualitatively
- use of the FDA’s ‘Market Watch’ and ‘Safety Alert’ paradigms for drugs may trigger a recall to prevent patients from a wider risk for GenAI-enabled devices
- monitor for exacerbation of disparities in access and use

**Figure 1:**
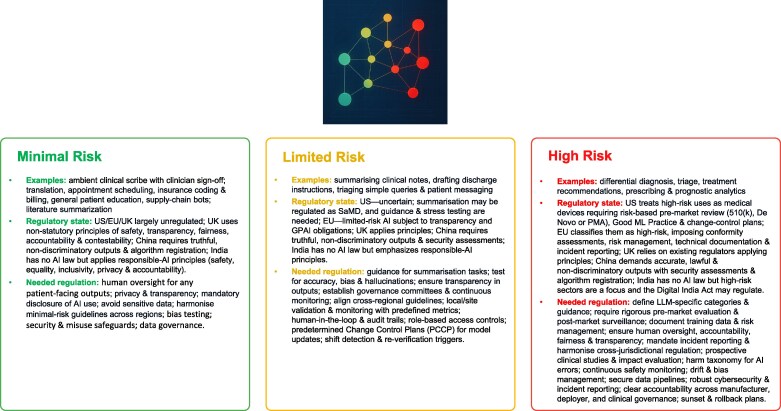
Functional risk tiers for generative AI in healthcare and their regulatory position. The figure groups GenAI uses into Minimal, Limited, and High risk. Minimal-risk examples are largely outside formal medical-device regimes in the US/EU/UK, with emphasis on non-statutory principles; needed safeguards include human oversight for any patient-facing text, bias testing, privacy/security controls, and data governance. Limited-risk tasks face mixed/uncertain treatment and may become SaMD when influencing care; needed measures include task-specific guidance, accuracy/calibration thresholds, predetermined change-control plans, and shift detection with re-verification. High-risk functions are generally regulated as medical devices (US/EU) or high-risk AI; needed measures include LLM-specific guidance, rigorous logging/traceability, defined human-oversight roles, change control, and reverting to the previous version of the software. (Classification depends on intended use and clinical context.) The critical link between the two regulations, the EU AI Act and the Medical Device Regulation (MDR), is the conformity assessment. Under Article 6(1) of the EU AI Act, an AI system is automatically classified as ‘High Risk’ if it pertains to a safety component of a product (or the product itself) covered by the MDR, and requires a third-party conformity assessment (involvement of a Notified Body). Specifically, these associations are as follows: 1. High Risk (EU AI Act), Associated MDR Classes: Class IIa, Class IIb, Class III; 2. Limited Risk (EU AI Act), Associated MDR Classes: Class I (Self-Certified) or Non-Device Software; 3. Minimal Risk (EU AI Act), Associated MDR Classes: Class I (Basic) or Operational Software (Non-Device).
US FDA: Clinical Decision Support Software Final Guidance (Oct 2022; https://content.govdelivery.com/accounts/USFDA/bulletins/32e8cb8);US FDA: PCCP Final Guidance (Dec 2024; https://www.fda.gov/regulatory-information/search-fda-guidance-documents/marketing-submission-recommendations-predetermined-change-control-plan-artificial-intelligence);US FDA: AI in SaMD (Mar 2025; https://www.fda.gov/medical-devices/software-medical-device-samd/artificial-intelligence-software-medical-device)UK MHRA: Software & AI as a Medical Device guidance (https://www.gov.uk/government/publications/software-and-artificial-intelligence-ai-as-a-medical-device/software-and-artificial-intelligence-ai-as-a-medical-device);UK MHRA: Change Programme roadmap (2023–2025; https://www.gov.uk/government/publications/software-and-ai-as-a-medical-device-change-programme/software-and-ai-as-a-medical-device-change-programme-roadmap)EU AI Act: high-risk obligations for medical devices (https://artificialintelligenceact.eu/article/16/)China: Do you have an “early days” generative AI strategy? - Chinese mainland and Hong Kong perspective (February 2024; https://www.pwccn.com/en/issues/generative-ai/are-you-ready-for-a-generative-ai-early-bird-strategyfeb2024.html);China: NMPA AI-SaMD classification & clinical evaluation guidelines (https://english.nmpa.gov.cn/2021-07/08/c_660267.htm)India: CDSCO Medical Devices Rules 2017 (https://cdsco.gov.in/opencms/resources/UploadCDSCOWeb/2022/m_device/Medical%20Devices%20Rules, %202017.pdf);India: ICMR Ethical AI Guidelines (2023; https://www.icmr.gov.in/ethical-guidelines-for-application-of-artificial-intelligence-in-biomedical-research-and-healthcare);India: Digital Personal Data Protection Act (2023; https://www.dpdpa.in/)Article 6(1) EU AI Act: Defines high-risk systems based on the requirement for third-party conformity assessment under harmonized EU legislation (like MDR). https://ai-act-law.eu/article/6/MDR Rule 11 (Annex VIII): The classification rule that pushes most medical AI software from Class I into Class IIa or higher, effectively making them ‘High Risk’ under the AI Act. https://www.medical-device-regulation.eu/2019/08/08/annex-viii/MDCG 2019-11: Guidance on Qualification and Classification of Software, confirming the up-classification of diagnostic software. https://health.ec.europa.eu/document/download/b45335c5-1679-4c71-a91c-fc7a4d37f12b_en

Risk management pertinent to GenAI-enabled devices draws from existing AI-enabled devices (***Figure 1*)**, and new risks pertaining to new intended uses or new applications in existing uses that have been enabled by GenAI, and what new control mechanisms (i.e. related to governance, training, feedback mechanisms, and real-world performance evaluation), may be needed to assess and mitigate these risks (**Box 5**).

Box 5 Recurrent Risks and Stage-Specific Mitigation Controls

**Table:** 

Recurrent Risk	Pre-market Objective (specify & verify)	Post-market Objective (monitor & correct)
**Stochastic Outputs**	Declare operating limits (e.g. partial AUROC in low-false-positive-rate region, subgroup calibration; document output variance/uncertainty reporting); validate on locked external sets; define guardrails and human-override points.	Instrument logging (prompts/contexts, seeds/decoding parameters, model version); require human sign-off for high-impact actions; track variance/instability signals; trigger monitor, detect, and correct, or rollback via PCCP if instability exceeds thresholds.
**Data Distribution Shift/Drift**	Stress-test on plausible shifts; pre-specify drift metrics, alert thresholds, and acceptance criteria for updates.	Run automated drift monitors; investigate alerts; apply threshold retuning or model/prompt updates under PCCP; document impact.
**Data Equity & Subgroup Performance**	Report subgroup metrics and calibration; include underrepresented cohorts in validation; set subgroup acceptance performance minima.	Audit subgroup dashboards; investigate disparities; apply targeted data augmentation or threshold adjustments; re-verify.
**Human oversight**	Define the human’s control function (block-list of non-actionable recommendations, checklists); verify human-factors claims.	Measure adherence (override rates, near-miss reviews); refine workflows/training; tighten non-actionable categories if needed.
**Predetermined Change Control Plans (PCCP)**	Pre-specify update triggers, locked tests, non-inferiority margins, and rollback criteria.	Execute updates only after passing benchmarks; surveil post-release; rollback if regression is detected.

Some risks are intrinsic to GenAI and require tailored controls. First, stochastic sampling and non-determinism require its safe use to hinge on declaring operating limits (e.g. variability bounds, uncertainty disclosure) and enforcing human monitoring for high-impact actions. Second, the prompt and context dependence, including retrieval-augmented generation (a pattern that combines search with generation so a model provides answers using external, cited sources rather than only its internal weights), creates a supply of content in which small changes of the prompts, the retrieval of the data, or the decoding parameters can shift clinical recommendations. Third, updates (e.g. model weights, prompt templates, etc) make change controls more frequent and multi-dimensional than in locked algorithms. Finally, provenance and auditability of generated content are central: regulators and hospitals need traceable links between inputs, prompts, parameters, and outputs to reproduce adverse events and support corrective actions (**Box 5**).

Other hazards are shared with traditional AI and can be addressed with the established TPLC practices, adapted where needed. Data representativeness and equitable access^24^ still determine whether performance generalizes across demographics and sites. Data distribution shift (due, for example, to changes in demographics, devices, workflows, etc) remains a leading cause of algorithm performance degradation, requiring pre-specified drift metrics and post-market monitoring. Opacity/interpretability continues to challenge clinical trust and error analysis, even if GenAI adds chain-of-thought–style rationales. Cybersecurity and human integration likewise prevent data leakage/manipulation, and well-designed oversight roles ensure that clinicians understand when to rely on, or override, AI output.

The EU’s AI Act Articles 10–16 set horizontal requirements that an AI-enabled device must present. These provisions make the aforementioned stochastic behavior of the GenAI algorithms more governable and auditable: (i) data handling and data governance require the training/validation/test datasets must be relevant to the intended context, appropriately representative, and managed to detect and mitigate bias (Article 10);^25^ (ii) technical documentation requires pre-market, living documentation that should be sufficient for authorities to assess compliance (Article 11, cross-referencing Annex IV items);^26^ (iii) record-keeping requires built-in event logging over the system lifecycle to enable traceability and post-market monitoring (Article 12);^27^ (iv) transparency to users requires both the limits of the required inputs and the provision of instructions that make outputs interpretable (Article 13);^28^ (v) human oversight requires the design of features and deployment controls that enable the monitoring, intervention, and safe override (Article 14);^29^ (vi) the accuracy, robustness, and cybersecurity assessment require the definition of metrics, and the robustness should be assessed throughout the lifecycle (Article 15).^30^ (vi) Article 16 allocates duties to providers and users, by ensuring conformity with Articles 10–15, and maintaining a quality management system (keeping documentation/logs, enabling the issuance of an EU Declaration of Conformity and affixing CE marking, as well as demonstrating compliance on request).^31^

Therefore, risk management involves the TPLC, which is a continuum of the traditional pre-market evaluation and post-market monitoring processes, as well as exploring yet unknown options that may require new authorities, recognising that post-market strategy risk assessment and mitigation starts with pre-market.

## Conclusions

With the emergence of GenAI, the close relationship between AI-driven decision-making systems and humans (of a wide variety and diversity of cultural beliefs) will inevitably become more complex in circumstances with multiple and possibly competing values, especially in the medical domain, where one meets vast inequalities even within the same country.^32,33^ The emergence of GenAI in the health policy domain makes these processes even more challenging when considering principles such as trustworthiness and ethical use of GenAI, aiming to promote beneficence, non-maleficence, and justice, and to ensure autonomy.

**Figure 2:**
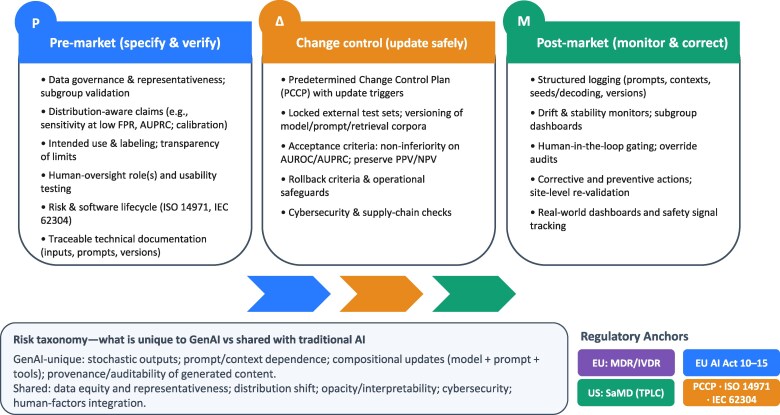
GenAI-adapted total product lifecycle (TPLC) for medical devices. Columns depict (1) Pre-market: data governance and subgroup validation; distribution-aware claims (e.g. sensitivity at low FPR, AUPRC, calibration); intended use/labelling and usability. (2) Change control: PCCP with update triggers; versioning of model/prompt sources; locked external tests; acceptance and rollback criteria. (3) Post-market: structured logging (prompts, contexts, seeds, versions), drift and subgroup dashboards, human-in-the-loop gating, corrective and preventive actions, site re-validation. Side taxonomy separates GenAI-unique vs. shared risks. Footer anchors: EU MDR/IVDR, AI Act (Arts 10–15), FDA SaMD TPLC, ISO 14971, IEC 62304. FPR: False positive rate; PPV: Positive predictive value; NPV: Negative predictive value; AUPRC: Area Under the Precision–Recall Curve; PCCP: Predetermined Change Control Plan.

We identify six priorities: (1) harmonized, distribution-aware performance metrics for GenAI; (2) minimum audit-logging schema (inputs/prompts/decoding/versioning) to support forensics and surveillance; (3) PCCP acceptance-testing templates for stochastic models; (4) site-level validation guidance for context-sensitive behavior; (5) integration pathways that align EU AI Act Articles 10–16 with Regulation (EU) 2017/745 (Medical Device Regulation**)** and Regulation (EU) 2017/7 (In Vitro Diagnostic Medical Devices Regulation) and FDA’s SaMD assessments, leveraging the harmonized frameworks and good machine learning practice principles established by the International Medical Device Regulators Forum (IMDRF) to ensure global interoperability; and (6) evidence standards for the design and evaluation of human-oversight workflows (***Figure 2***).

As the impact of national regulations will depend upon, and evolve through, interpretation, application, guidelines, definitions, exceptions, and potential amendments, developers, administrators and clinicians should continuously assess their obligations and invest in compliance measures with national regulators, prior to the development, implementation and deployment phases. Recognising the importance of these regulatory principles might play out in clinical practice, we now introduce two clinical vignettes in the Online Supplement.

The high-stakes GenAI environment clearly points to a situation where GenAI manufacturers will continue to have strong incentives for swift innovation processes, even if those come at the expense of safety. Thus, the existential threat resulting from the various policy frameworks, i.e. from the EU, US, UK, etc., may create a false sense of security, without paying enough attention to socioeconomic inequalities. Therefore, for GenAI in medicine to fully realize its transforming potential, it is imperative to address the ethical challenges head-on. This includes implementing, across country borders, robust data protection measures, actively combatting bias, ensuring system transparency, and establishing clear accountability structures. By doing so, the clinical community can responsibly harness the power of GenAI to usher in a new era of efficiency, safety, and precision care.^34–40^

**Figure 3:**
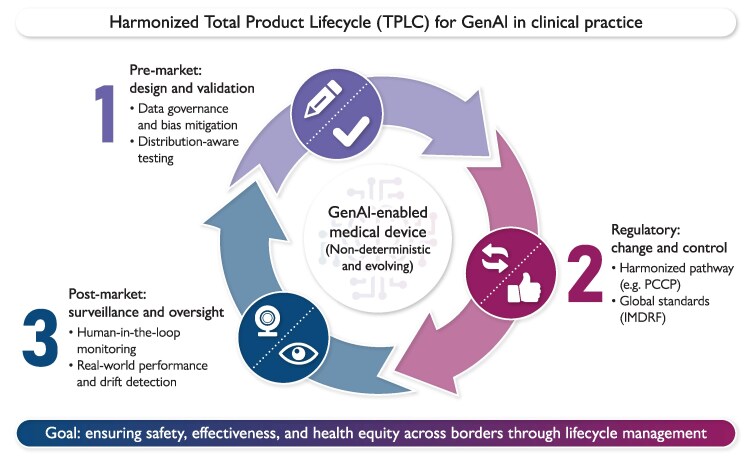
Central illustration. Harmonized Total Product Lifecycle (TPLC) for GenAI in Clinical Practice. Outline of a framework to manage non-deterministic, evolving GenAI models. It connects three key phases in a continuous loop: pre-market design focusing on bias mitigation and rigorous testing; regulatory control using adaptive pathways like predetermined change control plans (PCCP) and international standards; and post-market surveillance requiring human oversight and real-world performance monitoring. This continuous cycle ensures ongoing safety, effectiveness, and health equity across borders.

The established TPLC approach to regulating medical devices, including AI-enabled devices, incorporates technologies that are intended to iterate or change at a much faster pace than ever before, throughout their TPLC, or technologies that, in a sense, never represent a final ‘product’.^9,10,21^ Similarly, as GenAI enters clinical workflows, a new infrastructure for growth with guardrails is needed to be adopted, such that manufacturers can effectively devise a pre-market strategy and on-site monitor the post-market performance of their GenAI-enabled devices, in a manner that these devices continue to be safe and effective, throughout their TPLC, as they change (***Figure 3***). To achieve the transformational innovation and efficiency of GenAI benefits, organisations will be challenged to more fully embrace new digital technologies and invest resources in advanced data management capabilities, and internal monitoring and evaluation expertise.

Importantly, processes that increase patient-trust of GenAI-guided decisions may help reverse lower willingness and/or bias to follow a clinician’s advice when AI is believed to be involved in advice generation, even when AI is supposedly supervised by clinicians.^41^

## Supplementary Material

ztag019_Supplementary_Data

## Data Availability

No new data were generated or analysed in support of this research
