# Supplementary material for: Total product lifecycle regulatory considerations and recommendations for generative AI-enabled medical devices
Source: Eur Heart J Digit Health. 2026 Feb 3;7(3):ztag019. doi: 10.1093/ehjdh/ztag019 (PMC12959241; doi:10.1093/ehjdh/ztag019)
Supplement: ztag019_Supplementary_Data [file ztag019_supplementary_data.docx]

**Clinical Vignette: GenAI-assisted triage in chest pain, and how regulation upgrades performance**

**A 61-year-old woman with hypertension and hyperlipidemia presents to the ED 90 minutes after abrupt substernal pressure radiating to the left arm, now improved. Vitals are stable; pain 2/10. ECG shows borderline lateral ST-segment depression without contiguous changes. Initial high-sensitivity troponin is below the 99th-percentile cutoff; chest X-ray is unremarkable.**

On arrival, a generative-AI triage agent (large language model with retrieval to guideline snippets and site protocols) ingests EHR data, the raw ECG trace, and nurse triage notes. It produces a structured, human-readable brief:

- **Risk synthesis:** HEART ≈ 5 (history moderate, ECG nonspecific, age > 60, risk factors ≥ 2, initial troponin low).
- **Top differentials:** NSTE-ACS > demand ischemia > GERD.
- **Action plan:** “0/1-hour hs-cTn pathway; continuous telemetry; consider CT-coronary angiography (CTCA) if serial troponins negative; avoid early discharge unless delta < rule-out threshold and HEART ≤ 3.”
- **Rationale & caveats:** cites local protocol and ESC/ACC excerpts; flags that ECG noise could mask subtle ST-T changes and that symptom offset does not exclude ACS.

Because the generative model is stochastic, an ensemble is run with conservative decoding; a policy guardrail requires concordance on “safe discharge.” In this case, one candidate output suggested discharge to rapid outpatient testing; the guardrail blocks it (HEART > 3) and prompts clinician review. Serial troponins at 1 and 3 hours show a small but significant rise; CTCA reveals a proximal LAD stenosis with high-risk plaque features. The patient proceeds to early invasive angiography and stenting, with uneventful recovery.

How regulation improves performance (within this case)

Modern frameworks (e.g., EU AI Act requirements on data governance, documentation, logging, transparency, human oversight, and accuracy/robustness; FDA’s SaMD lifecycle with a Predetermined Change Control Plan; and harmonized standards for risk management and software quality) drive concrete design choices that *raise clinical performance and safety*:

1. **Data & bias governance → better sensitivity across subgroups.** Curated, documented training/validation sets with subgroup analyses (e.g., women with atypical ACS) reduce under-diagnosis and enforce **declared operating points** (e.g., ≥99% sensitivity for rule-out pathways).
2. **Transparency & intended-use labeling → correct operating conditions.** The model’s label specifies the triage setting, inputs required (raw ECG + hs-cTn), and the **performance envelope** (e.g., partial AUROC in the low-FPR regime, calibration targets), lowering misuse and off-label deployment that degrade PPV/NPV.
3. **Human oversight as a control function → fewer unsafe discharges.** Guardrails make certain recommendations (e.g., discharge in moderate/high risk) **non-actionable without clinician sign-off**, reducing false-negative triage.
4. **Built-in logging/traceability → faster error correction and calibration.** Automatic capture of prompts, context, decoding parameters/seeds, and intermediate rationales enables reproducible case review, root-cause analysis, and continuous recalibration (e.g., troponin-delta thresholds) without performance drift.
5. **Accuracy/robustness & cybersecurity testing → resilience to real-world noise.** Mandatory stress tests (ECG motion artifact, lead misplacement, timestamp mismatches) and adversarial/abuse-input checks improve robustness, preserving discrimination and calibration under ED conditions.
6. **Change control (PCCP) → updates without regressions.** Any model or prompt-template update must pass pre-specified acceptance criteria on locked external test sets and post-market performance monitors, preventing silent degradation after deployment.
7. **Post-market surveillance → learning health-system loop.** Real-world performance dashboards (e.g., monthly AUROC/AUPRC, sensitivity at clinical operating points, subgroup calibration) trigger corrective actions, threshold re-tuning, or rollback—sustaining performance over time.

What this illustrates: GenAI adds value by *synthesizing* multimodal signals; regulations make that value dependable—shifting practice from one-off accuracy claims to distribution-aware, logged, monitored, and change-controlled performance within a total product lifecycle.

**Clinical workflow abbreviations**

- ED — Emergency Department.
- EHR — Electronic Health Record.
- ECG — Electrocardiogram (heart’s electrical tracing).
- ST (segment) — Not an acronym; ECG segment reflecting early ventricular repolarization.
- hs-cTn — High-sensitivity cardiac troponin (biomarker of myocardial injury).
- HEART (score) — **H**istory, **E**CG, **A**ge, **R**isk factors, **T**roponin; 0–10 chest-pain risk score.
- CTCA — Computed Tomography Coronary Angiography (non-invasive coronary imaging).
- ACS — Acute Coronary Syndrome (MI/unstable angina spectrum).
- NSTE-ACS — Non–ST-Elevation Acute Coronary Syndrome.
- LAD — Left Anterior Descending (coronary artery).
- GERD — Gastroesophageal Reflux Disease.

**Regulatory quality abbreviations**

- EU AI Act — European Union Artificial Intelligence Act (horizontal AI regulation).
- FDA — U.S. Food and Drug Administration.
- SaMD — Software as a Medical Device (regulated clinical software).
- PCCP — Predetermined Change Control Plan (pre-specified rules/tests for future updates).

**Model evaluation metrics abbreviations**

- AUROC — Area Under the Receiver Operating Characteristic curve (threshold-free ranking metric).
- AUPRC — Area Under the Precision–Recall Curve (useful for class imbalance).
- FPR — False Positive Rate = FP/(FP+TN).
- PPV — Positive Predictive Value = TP/(TP+FP).
- NPV — Negative Predictive Value = TN/(TN+FN).

*(Terms like “delta” = change over time and “telemetry” = continuous rhythm monitoring are not acronyms but appear in the text.)*

**Clinical Vignette: GenAI-assisted CTPA report with radiologist gatekeeping (and how regulation upgrades performance)**

**A 72-year-old woman with recent hip surgery presents with sudden dyspnea and pleuritic chest pain. The ED orders CTPA. As images land in PACS (via DICOM), a validated GenAI service auto-launches.**

1. **Model analysis → draft report**

The model detects multiple left lower-lobe segmental filling defects, quantifies clot burden, estimates RV/LV ratio ≈ 1.1, and flags small peripheral wedge opacities. It composes **a structured draft** (Technique/Findings/Impression), inserts confidence statements (“moderate confidence; mild respiratory motion”), highlights key slices, and embeds DICOM-SR measurements.

**Regulatory lever → better inputs & claims.** Data-governance requirements (EU AI Act Art.10; SaMD QMS) enforce curated, documented training/validation with **subgroup performance** (e.g., postoperative patients), declared **intended use** (ED triage CTPA), and operating points (e.g., sensitivity at low FPR). This reduces hidden bias and mis-use and improves real-world sensitivity/specificity.

1. **Automated completeness & safety checks (pre-review)**

Deterministic validators confirm presence of required sections, laterality consistency, explicit hemorrhage/effusion statements, and **critical-result** language if PE is asserted. Gaps are auto-filled before any human sees the draft.

**Regulatory lever → transparency & traceability.** Articles 11–13 and MDR/IVDR-style documentation/logging require **full provenance** (model/version, prompt template, decoding parameters/seed, input list), enabling audit trails and reproducibility; harmonized formats (DICOM-SR) support structured QA.

1. **Radiologist review (accuracy & completeness gate)**

In the **RIS** viewer, the on-call radiologist inspects overlays and uncertainty notes, upgrades extent to lobar PE with mild RV strain, adds ancillary findings (small pleural effusion), and edits the impression to include anticoagulation recommendation and **time-stamped critical communication.** Only after sign-off does the report flow to the **EHR** and trigger PE pathway orders.

**Regulatory lever → human oversight that improves safety.** Human-in-the-loop controls (EU AI Act Art.14; SaMD human-factors) make high-impact suggestions **non-actionable** until a credentialed reader approves; this reduces false-negative discharges and standardizes reporting via checklists/templates.

1. **Change control & robustness**

A week later, the vendor ships an update to motion-artifact handling. Because a **PCCP** exists, the site auto-tests on a locked external set (including high-motion cases) with pre-specified acceptance criteria (non-inferior AUROC/AUPRC and maintained PPV at clinical operating points). The update proceeds only after passing.

**Regulatory lever → no silent regressions.** Predetermined change plans, risk management (ISO 14971), software lifecycle (IEC 62304), and cybersecurity testing require **pre-deployment gates** that protect discrimination and calibration under real ED conditions.

1. **Post-market surveillance & learning**

The system logs AI draft ↔ final report diffs, timestamps, and outcomes. A dashboard tracks monthly sensitivity for lobar/segmental PE, false-positive rates in COPD, and subgroup calibration. Drift triggers prompt-template tweaks or rollback.

**Regulatory lever → performance that stays good.** Articles 12 & 15 and TPLC obligations require **continuous monitoring** and corrective actions; distribution-aware metrics and subgroup audits keep performance stable across time and sites.

**Outcome.** The patient starts anticoagulation and is admitted. The final signed report—**not** the AI draft—enters the record; all artifacts are retained for QA and regulator-ready audits.

**Clinical & imaging abbreviations**

- **AI** — Artificial Intelligence.
- **GenAI** — Generative Artificial Intelligence (models that compose new text/images).
- **CT** — Computed Tomography.
- **MRI** — Magnetic Resonance Imaging.
- **CTPA** — CT Pulmonary Angiography (contrast CT to detect pulmonary emboli).
- **PE** — Pulmonary Embolism (clot in pulmonary arteries).
- **ED** — Emergency Department.
- **RV/LV ratio** — Right-ventricle to left-ventricle diameter ratio on CT; a marker of right-heart strain.

**Radiology IT & data standards abbreviations**

- **PACS** — Picture Archiving and Communication System (stores/displays images).
- **DICOM** — Digital Imaging and Communications in Medicine (file/network standard for medical images).
- **DICOM-SR** — DICOM Structured Reporting (standardized, machine-readable report objects).
- **RIS** — Radiology Information System (worklists, reporting, scheduling).
- **EHR** — Electronic Health Record (longitudinal patient record).

**Regulatory, quality, and lifecycle abbreviations**

- **QMS** — Quality Management System (organization-wide processes for design, testing, release, CAPA).
- **SaMD** — Software as a Medical Device (clinical software regulated as a device).
- **EU AI Act** — European Union Artificial Intelligence Act (horizontal AI regulation).
- **MDR / IVDR** — EU Medical Device Regulation / In Vitro Diagnostic Regulation.
- **PCCP** — Predetermined Change Control Plan (pre-specified tests/criteria for safe model updates).
- **TPLC** — Total Product Life Cycle (end-to-end oversight: pre-market → post-market).
- **ISO 14971** — International standard for medical-device risk management.
- **IEC 62304** — International standard for medical-device software lifecycle processes.
- **QA** — Quality Assurance (systematic activities ensuring requirements are met).

**Evaluation metrics (used in acceptance criteria & monitoring) abbreviations**

- **AUROC** — Area Under the Receiver Operating Characteristic curve; probability a random positive scores higher than a random negative.
- **AUPRC** — Area Under the Precision–Recall Curve; informative under class imbalance.
- **PPV** — Positive Predictive Value = TP/(TP+FP).
- **FPR** — False Positive Rate = FP/(FP+TN).

**Comorbidities & cohorts (mentioned in monitoring) abbreviations**

- **COPD** — Chronic Obstructive Pulmonary Disease.

(TP = true positives, FP = false positives, TN = true negatives.)
